# Implementation of a Multimodal Knowledge-Exchange Platform to Provide Trauma Critical Care Education During the Ongoing Conflict in Ukraine

**DOI:** 10.1001/jamanetworkopen.2023.0050

**Published:** 2023-02-10

**Authors:** Lucrezia Rovati, Simon Zec, Dmytro Dziuba, Anna Masoodi, Aysun Tekin, Claudia Castillo Zambrano, Meghan Brown, Oleksiy Khavryuchenko, Oleksandr Bugay, Grygorii Khytryi, Oleg Loskutov, Yue Dong, Ognjen Gajic, Alexander S. Niven

**Affiliations:** 1 Division of Pulmonary and Critical Care Medicine, Department of Medicine, Mayo Clinic, Rochester, Minnesota; 2 School of Medicine and Surgery, University of Milano-Bicocca, Milan, Italy; 3 Department of Anesthesia, Critical Care and Pain Medicine, Beth Israel Deaconess Medical Center, Boston, Massachusetts; 4 Department of Anesthesiology and Intensive Care, Shupyk University of Public Health of Ukraine, Kyiv, Ukraine; 5 Division of Nephrology and Hypertension, Department of Medicine, Mayo Clinic, Rochester, Minnesota; 6 National Military Medical Clinical Center, Main Military Clinical Hospital, Kyiv, Ukraine; 7 Ukrainian Military Medical Academy, Kyiv, Ukraine; 8 Department of Anesthesiology and Perioperative Medicine, Mayo Clinic, Rochester, Minnesota

## Abstract

**Importance:**

The conflict in Ukraine has forced civilian hospitals with limited trauma and battlefield medicine experience to care for casualties of war, placing significant strain on the health care system. Using the Checklist for Early Recognition and Treatment of Acute Illness and Injury (CERTAIN) program, a multimodal trauma critical care knowledge-exchange platform was created for clinicians practicing in these institutions.

**Objectives:**

To describe the development and implementation of the CERTAIN for Ukraine program and to evaluate the reach of this intervention, together with participant engagement and satisfaction.

**Design, Setting, and Participants:**

This quality improvement study included clinicians caring for critically ill patients during the ongoing Ukrainian conflict who were part of a community developed using a messaging app. The program was implemented by a group of international trauma and critical care experts in collaboration with critical care leaders from the Shupyk National Healthcare University in Kyiv, Ukraine. This study evaluates data collected from the CERTAIN for Ukraine program from its launch on April 9, 2022, to August 31, 2022.

**Interventions:**

The initiative comprised a longitudinal series of interactive tele-education sessions, a webpage containing the CERTAIN approach and current trauma critical care guidelines translated into Ukrainian and Russian, and a private messaging chat for asynchronous discussion.

**Main Outcomes and Measures:**

Participant engagement and satisfaction were tracked using multimedia analytics and a post-session survey.

**Results:**

Since program launch, 838 participants have joined the messaging group, and 6 tele-education sessions have been delivered, with 1835 total views. The CERTAIN website has had 3527 visits, mainly from Ukraine (1378 [39%]) and the United States (1060 [30%]). Of the 74 completed postsession surveys, 65 respondents (88%) rated the course content excellent or very good, and 73 (99%) recommended it to others.

**Conclusions and Relevance:**

The findings of this quality improvement study indicate that, using widely available and low-cost platforms, knowledge was shared rapidly and efficiently to a large community of clinicians practicing in a wartime environment with broad-based engagement and a high level of learner satisfaction.

## Key Points

**Question:**

Can trauma critical care education be provided to clinicians practicing in a wartime environment via widely available, low-cost platforms?

**Findings:**

In this quality improvement study with 838 participants performed during the ongoing conflict in Ukraine, a longitudinal series of interactive tele-education sessions supplemented by online resources and a secure social media chat group provided rapid delivery of trauma critical care education with high participant engagement and satisfaction.

**Meaning:**

These findings suggest that simple tele-education interventions can rapidly and efficiently share best practices in the care of critically ill wartime casualties to a large community of clinicians practicing in an area of active conflict.

## Teaser

This quality improvement study describes the development and implementation of a multimodal trauma critical care knowledge-exchange platform during the ongoing conflict in Ukraine and evaluates the reach of this intervention, together with participant engagement and satisfaction.

## Introduction

The conflict in Ukraine has posed an unpreceded strain on local and regional health care resources since fighting began on February 24, 2022, adding to the significant ongoing challenges created by the COVID-19 pandemic.^1^ As of November 27, 2022, in addition to the number of undisclosed military causalities, the Office of the United Nations High Commissioner for Human Rights had recorded 6655 civilian deaths and 10 368 injuries, mainly due to the use of explosive weapons with wide area effects.^2^ Since the beginning of the conflict, the World Health Organization (WHO) has reported 715 attacks on health care facilities that have resulted in 129 injuries and 100 fatalities.^3^ Clinicians working in such conditions are also facing shortages of staff and supplies, destruction of infrastructure, burnout, and anxiety.^4^ Many civilian hospitals with limited trauma and battlefield medicine experience are by necessity caring for large numbers of injured civilian and military patients, staffed by clinicians who are unaccustomed to the management of multiple trauma and other war-related injuries.^5^

Over the past several years, telemedicine has proven to be an effective disaster response resource to support populations in need.^6^ Tele–critical care and tele-consultation services have been implemented in different wartime scenarios.^7,8,9^ In addition, remote education programs can offer a safe, feasible, and rapid way to distribute knowledge and provide support to areas of active conflict.^10,11^ These methods have been shown to be effective in improving quality of care and patient outcomes in intensive care units (ICUs) where clinicians have limited critical care subspecialty training and experience.^12^ Barriers to effective implementation of international tele-consultation and tele-education programs include language differences and technical, cultural, and training issues, including the complex, expensive technology infrastructure often used in these programs.^13,14^ Simpler approaches using commonly available technology and social media resources have been used for tele-education and clinical support during the COVID-19 pandemic among national and international health care collaborations.^15,16,17,18^ However, the generalizability of these approaches to combat conditions and their possible impact on the quality of care and disease outcomes in critically ill trauma patients is unknown.

Recognizing the acute need for additional resources, our group developed a multimodal knowledge-sharing platform to provide trauma, critical care, and disaster medicine education and clinical support for clinicians working in Ukraine during the ongoing conflict. This initiative, designed using the Analysis, Design, Development, Implementation, and Evaluation instruction design model, was based on the Checklist for Early Recognition and Treatment of Acute Illness and Injury (CERTAIN) program, a global ICU best practices, education, and quality improvement initiative.^19,20^ The aim of this study was to describe the development and implementation of the CERTAIN for Ukraine program and to evaluate the reach of this intervention, together with participant engagement and satisfaction.

## Methods

### Design

The protocol of this study was evaluated and approved by both the Mayo Clinic and Shupyk National Healthcare University institutional review boards, and a waiver of informed consent was granted because of the use of deidentified data. No patient names or other personal identifiers were shared within or outside of the secure messaging group or during the tele-education sessions. The images used for case discussions were free of identifying characteristics. Individual learner names and information were not collected. Results are reported following the Standards for Quality Improvement Reporting Excellence (SQUIRE) reporting guidelines.

Within 2 months of the beginning of the Ukrainian conflict, a group of international trauma and critical care experts in collaboration with the Shupyk National Healthcare University in Kyiv, Ukraine, created a multimodal knowledge-sharing platform for clinicians caring for critically ill patients. This quality improvement study evaluated initial data collected from this initiative from its launch on April 9 to August 31, 2022.

### The CERTAIN for Ukraine Program

Viber (Rakuten, Inc) secure messaging service was used to connect clinicians participating in this initiative, enabling them to ask general clinical questions and exchange educational material via a private chat group on an on-demand, asynchronous basis. Health care professionals were invited to participate in the community via an email shared via the Ukrainian Society of Anesthesiology and Intensive Care mailing list on April 21, 2022. Clinicians had the ability to invite other participants by word of mouth and referral weblinks.

Community information and available resources, including checklists, guidelines, and recommendations for the management of critically ill, multiorgan trauma patients, were gathered on the CERTAIN for Ukraine public webpage^21^ and translated into Ukrainian and Russian. The Society of Critical Care Medicine (SCCM) also provided administrative support and free access to translated SCCM resources.

**Figure 1.**
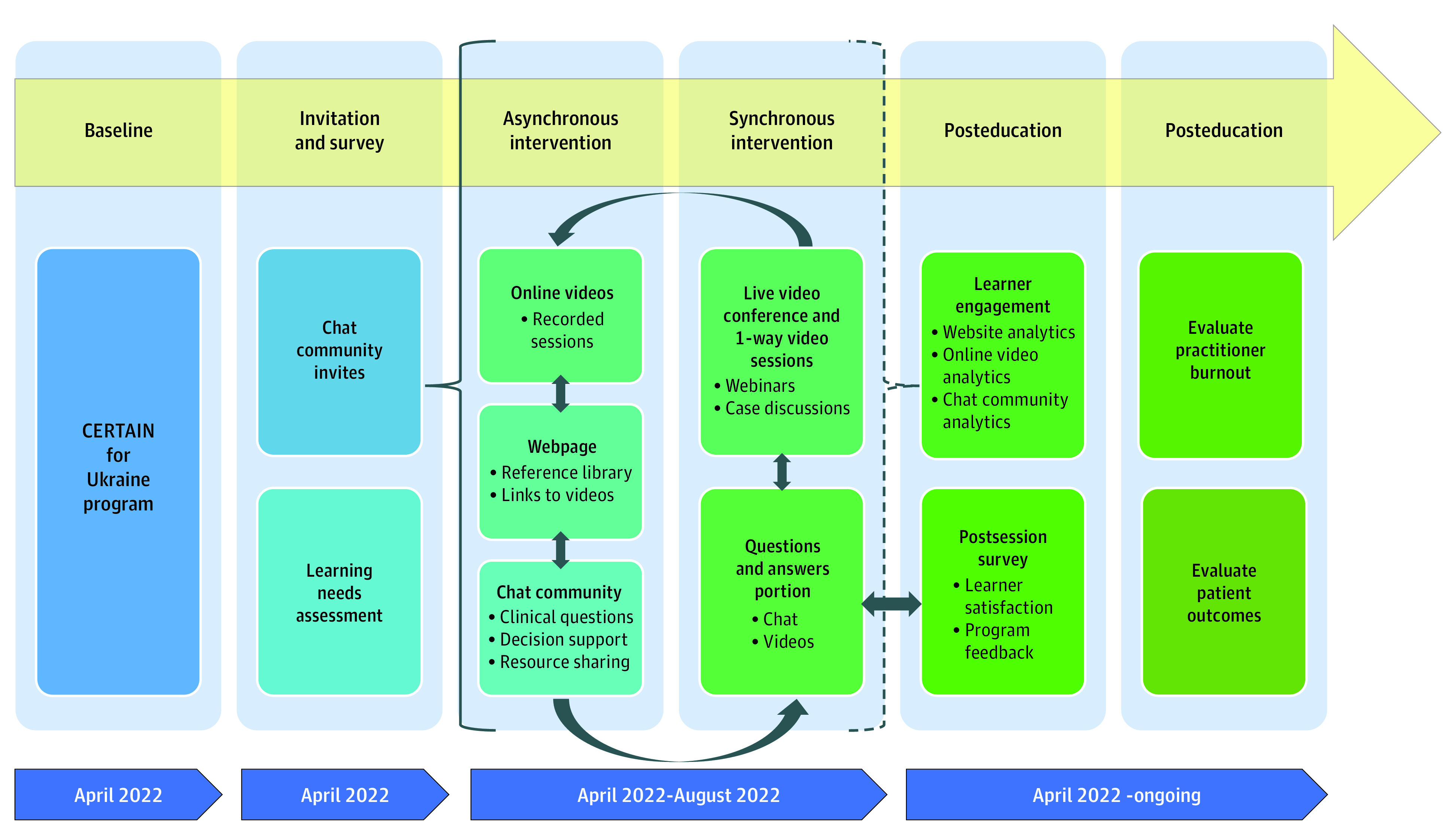
Project Outline for the Checklist for Early Recognition and Treatment of Acute Illness and Injury (CERTAIN) for Ukraine Program

The main tele-education intervention consisted of a series of case discussions and webinars on established approaches to battlefield trauma and critical care, held by a mixed faculty of expert intensivists, surgeons, emergency physicians, and anesthesiologists from the United States, United Kingdom, and Ukraine. The presentation topics were selected using a needs assessment survey completed by participating Ukraine clinicians (eFigure in Supplement 1) and were delivered using the Zoom video conferencing platform (Zoom Video Communication). These sessions were simultaneously livestreamed to an unlisted YouTube channel accessible through a link provided via the messaging group, and viewers could ask questions in real-time via either platform. These interactive sessions were recorded and stored on the project webpage to allow asynchronous viewing and further discussion on the group chat. Synchronous interpreter services were provided as subtitles during sessions by bilingual faculty and support staff, who also managed the group chat community discussions, translations, and content sharing. A schematization of the CERTAIN for Ukraine project is outlined in Figure 1.

### Outcome Measures

Learner engagement was assessed via analytics from the program website, the video sharing platform, and the messaging app. Metrics included number of website visits by country, city, and device type; number of participants and total messages within the messaging community; and number of live and on-demand views and total and average watch time on the video sharing platform. To assess learner needs, satisfaction, and feedback for program improvement, group participants were invited to complete an anonymous survey before the first and second live tele-education intervention and after each webinar (eFigure and eTable in Supplement 1).

### Statistical Analysis

Descriptive statistics were reported as numbers and percentages or means with standard deviations as appropriate. Excel version 2207 (Microsoft Corp) was used to conduct analyses.

## Results

The messaging group was established on April 9, 2022, and by August 31, 2022, 838 participants had joined the secure chat. During the same time period, 526 total messages were exchanged on the group. The month of May included the most messages (234 [44.5%]). Most messages included information and questions regarding the webinar discussions, clinical questions and cases, and links to openly available online educational resources, such as scientific articles, books, guidelines, and lectures.

**Figure 2.**
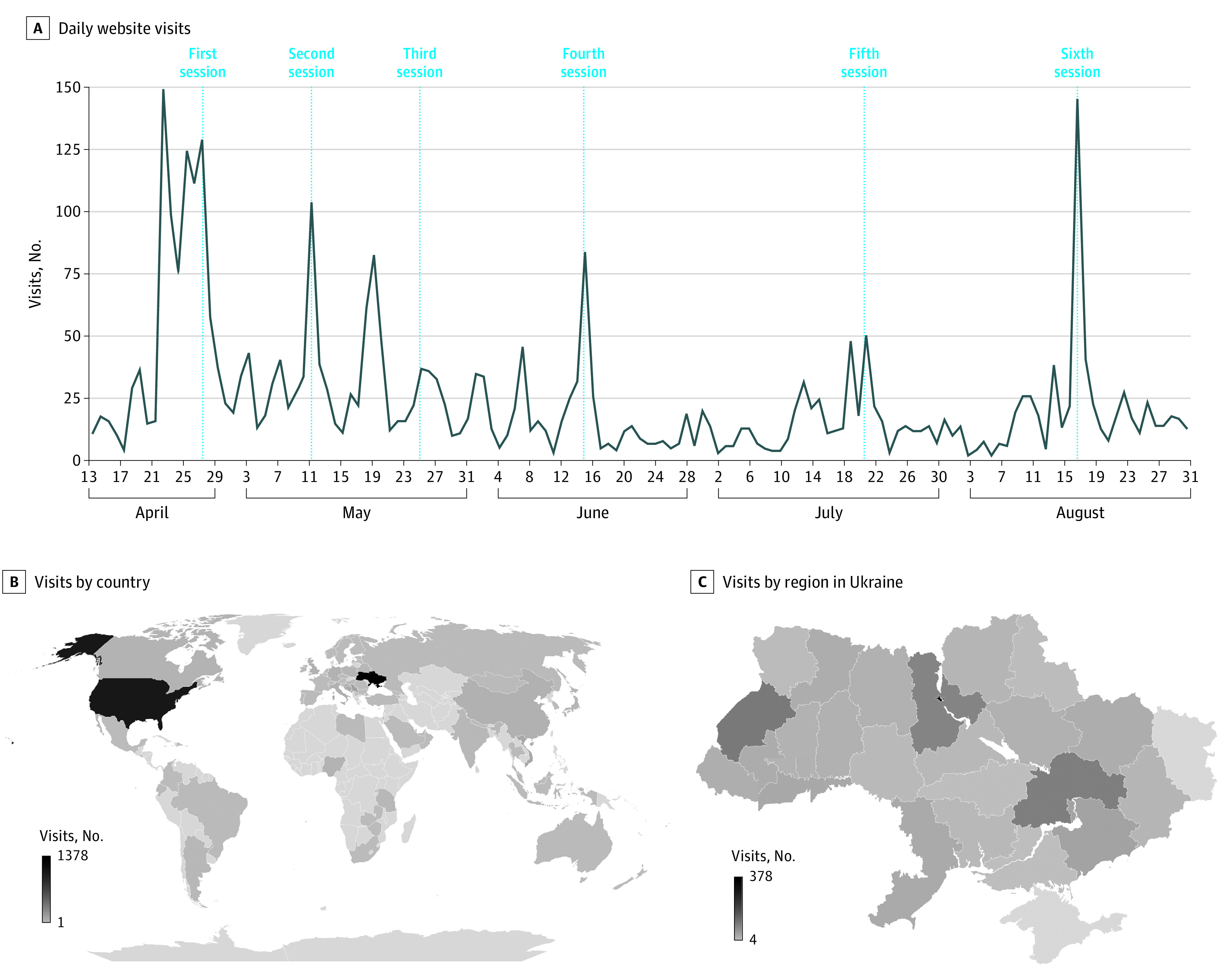
Checklist for Early Recognition and Treatment of Acute Illness and Injury Website Analytics A, Number of daily website visits and tele-education sessions. B, Website visits by country. C, Website visits by Ukraine region. Data were obtained from website hosting platform’s analytics.

The CERTAIN for Ukraine public webpage was launched on April 13.^21^ From the webpage launch to August 31, 2022, the CERTAIN website had 3527 visits by 2646 unique visitors, with the Ukraine webpage accounting for 2658 page views (Figure 2). The website visits were mainly from Ukraine (1378 [39.1%]), particularly from Kyiv, followed by L’viv and Dnipropetrovs’k. The second highest number of visitors were from the United States (1060 [30.1%]) (Figure 2). Most visits to the website were through mobile devices (2107 [59.7%]), followed by desktop computers (1394 [39.5%]). The source of these visits was primarily through direct links (2736 [77.6%]), followed by social media platforms (363 [10.3%]) and search engines (341 [9.7%]). Traffic spikes were recorded on days when tele-education sessions were conducted (Figure 2).

Six tele-education sessions were conducted on the video conferencing platform during the initial study period and broadcast on the video sharing platform on April 27, May 11 and 25, June 15, July 21, and August 17, 2022 (Table 1). These sessions included cases presented by Ukrainian clinicians using the CERTAIN case template, followed by multidisciplinary discussion of evidence-based clinical management strategies by Ukrainian and participating international faculty. Focused webinar presentations on topics selected from the needs assessment survey results were also prepared and delivered by this faculty group, with initial focus on trauma assessment and resuscitation, massive transfusion, blast injury, traumatic brain injury, sepsis, and acute respiratory distress syndrome (eFigure in Supplement 1). Tele-education session engagement on the video sharing platform is summarized in Table 1. The total number of views was 1835, with a mean (SD) view duration of 15:21 (2:11) minutes. For all sessions, most visits were through mobile devices (between 64% and 82% of total visits, per each webinar session).

**Table 1.** Analytics for the Tele-Education Sessions

Tele-education session No.	Date	Topic	No.			Watch time, h	Average view duration, min
			Peak concurrent live views	On-demand views	Unique viewers		
1	April 27, 2022	A systematic approach to trauma critical care and disaster medicine	52	716	508	93.9	11:04
2	May 11, 2022	The current state of medical care for the wounded during the war in Ukraine	28	323	226	62.6	16:38
3	May 25, 2022	Massive transfusion in trauma	25	241	176	44.8	15:16
4	June 15, 2022	Interactive case discussion	23	145	105	30.4	17:21
5	July 21, 2022	Preventing combat wound–related infections	12	129	80	18.9	14:08
6	August 17, 2022	Mechanical ventilation for ARDS patients	42	281	202	59.5	17:40

Abbreviation: ARDS, acute respiratory distress syndrome.

Postsession feedback was limited, with 74 survey responses. Response rate could not be computed due to survey dissemination using the group messaging app, where participants can join and leave over time, leading to a variable denominator. Most participants were attending physicians, followed by resident or fellow physicians. Survey results indicated a high degree of learner satisfaction, with 65 (88%) rating the course excellent or very good, and 73 (99%) stating that they would recommend this activity to others. Postsession survey results are summarized in Table 2 and the eTable in Supplement 1.

**Table 2.** Postsession Survey Results

Survey questions	Responses, No. (%)					
	Tele-education session date					Overall (n = 74)
	April 27, 2022 (n = 14)	May 11, 2022 (n = 39)	May 25, 2022 (n = 10)	July 21, 2022 (n = 4)	August 17, 2022 (n = 7)	
1. Identify your profession						
Resident or fellow physician	3 (21)	16 (41)	3 (30)	1 (25)	2 (29)	25 (34)
Attending physician	11 (79)	13 (33)	5 (50)	2 (50)	4 (57)	35 (47)
Health care executive	0	1 (3)	1 (10)	1 (25)	0	3 (4)
Other	0	9 (23)	1 (10)	0	1 (14)	11 (15)
2. How would you rate the online course contents?						
Poor	0	0	0	0	0	0
Fair	0	0	0	0	0	0
Good	1 (7)	6 (15)	0	1 (25)	1 (14)	9 (12)
Very good	7 (50)	10 (26)	4 (40)	0	1 (14)	22 (30)
Excellent	6 (43)	23 (59)	6 (60)	3 (75)	5 (72)	43 (58)
3. Was the course length too long, too short, or about right?						
Too long	2 (14)	3 (8)	1 (10)	0	0	6 (8)
About right	11 (79)	34 (87)	9 (90)	4 (100)	7 (100)	65 (88)
Too short	1 (7)	2 (5)	0	0	0	3 (4)
4. Would I recommend this activity to others?						
Yes	13 (93)	39 (100)	10 (100)	4 (100)	7 (100)	73 (99)
No	1 (7)	0	0	0	0	1 (1)

## Discussion

We report the development and implementation of the CERTAIN for Ukraine initiative, a multimodal knowledge-exchange platform designed to provide battlefield trauma and critical care education and clinical support to clinicians during the ongoing conflict in Ukraine. Since the beginning of ongoing hostilities, Ukrainian clinicians not formally trained in battlefield medicine have been faced with the challenge of synthesizing best practice recommendations and practical guidelines and quickly applying them at the bedside.^22^ Free and easy access to accurate clinical information provided by reputable organizations and medical societies becomes even more relevant during such circumstances of hybrid warfare and widespread misinformation. An integrated and structured approach is needed to avoid delays in implementation of evidence-based care delivery in this and similar environments. In particular, the WHO and other organizations have asked for the implementation of educational interventions using telemedicine, simulation, and internet-based training for clinicians working in a wartime setting.^23^

The CERTAIN approach has been previously shown to be associated with improving adherence with daily care processes, shortening length of stay, and decreasing mortality in variably resourced ICU settings.^19^ Moreover, a modification of the CERTAIN remote simulation training program, delivered using internet-based web resources and a social media platform, has been successfully applied during the COVID-19 pandemic to provide rapid knowledge exchange among clinicians in southeastern Europe and the United States.^15,16^ In this study, we have applied a further modification of the CERTAIN program to meet the specific needs of clinicians caring for combat injuries in Ukraine. In particular, this program was designed to provide knowledge and bedside decision support for the care of critically ill, multiorgan trauma patients via simple tele-education interventions.

In this program, we used readily available, low-cost video conference and social media platforms to share knowledge quickly and efficiently. Our data show that engagement was high, and our participants were satisfied with the program. Web page views and chat interactions were highest during the first weeks following the program launch and around the tele-education sessions. The reach of this intervention was broad, with website viewers not only from Ukraine and the United States but also from other countries both in Europe and all over the world (Figure 2). Website and video sharing platform visits were mainly conducted through mobile devices, underlying the importance of easy access to readily available technology platforms.

Previous experiences in delivering tele-education in other conflicts and postconflict settings, including Iraq, Syria, and Bosnia and Herzegovina, have shown the feasibility and efficacy of this approach.^6,10,11,12^ Tele-ICU programs have also been implemented in Syria with success.^7,8^ Other studies have shown how social media can be used by clinicians as educational support tools and for the delivery of tele-consultation services.^9,24,25^ In particular, during the COVID-19 pandemic, social media use has accelerated to the point of becoming an ubiquitous part of modern health care systems.^15,26^ These findings, in addition to our own experience, suggest that tele-education interventions offer a safe and effective option for training and clinical support during global emergencies.

To our knowledge, our study describes the first intervention attempting to provide tele-education services during the ongoing conflict in Ukraine. Our use of the CERTAIN program, a well-established method to teach a standardized, structured, and systematic approach for the evaluation of critically ill patients, allowed us to rapidly implement this tele-education intervention for Ukrainian clinicians with a high degree of engagement and satisfaction. Another strength of this program was the use of widely available and low-cost social media platforms to deliver this content to a large community of participants. In addition, the possibility of asynchronous viewing of tele-education sessions and continuous, on-demand communication via the group messaging chat with colleagues and experts facilitated participation. Lastly, providing synchronous interpretation in Ukrainian and translation of available resources helped increase the engagement of local, non-English speaking participants.

### Limitations

Our study has several limitations. Time constraints for Ukrainian clinicians imposed a barrier to the implementation of this program. To attenuate this problem, we organized our tele-education sessions according to the needs and feedback of our participants, limiting them to 1-hour sessions held 2 to 5 weeks apart and delivered after normal work hours in Ukraine, with asynchronous viewing options. However, mean view duration on the video sharing platform was still low, underlining the need for providing short education sessions and concise decision support tools that can be easily accessed by mobile devices. Another limitation of this program was that it was mainly attended by physicians, with no nursing and other health care professional (eg, respiratory therapist, pharmacist) involvement. Moreover, the postsession survey was completed by a small number of participants, and it was not possible to evaluate the response rate since the survey was shared on the group messaging app, which has a time-varying number of participants. Additionally in the current study, we did not evaluate the implementation effectiveness of this educational intervention and its impact on patient care and on the clinicians delivering it. Indeed, only the outcomes related to level 1 (reaction) of the Kirkpatrick 4-level training evaluation model are reported in this work.^27^ The chaotic and rapidly changing environment that the participating clinicians faced in their practice was deemed prohibitive at this time to a more rigorous evaluation of additional results of this training program. Further studies are needed to assess the impact of this project on important process and outcomes measures.

## Conclusions

This study found that a multimodal intervention to provide education and clinical support for the care of critically ill trauma patients in response to the conflict in Ukraine was feasible, inexpensive, and associated with a high degree of clinician engagement and satisfaction. This approach can be used as a model for the development of further education and quality improvement interventions in remote and austere environments during global emergencies and disasters.

## Notes

Supplement 1. **eFigure. **Learning Needs Assessment Survey Results **eTable. **Postsession Survey Additional Questions

Supplement 2.
Data Sharing Statement
