# Supplementary material for: Implementation of a Multimodal Knowledge-Exchange Platform to Provide Trauma Critical Care Education During the Ongoing Conflict in Ukraine
Source: JAMA Netw Open. 2023 Feb 10;6(2):e230050. doi: 10.1001/jamanetworkopen.2023.0050 (PMC9918882; doi:10.1001/jamanetworkopen.2023.0050)
Supplement: Supplement 1. — eFigure. Learning Needs Assessment Survey Results eTable. Postsession Survey Additional Questions [file jamanetwopen-e230050-s001.pdf]

Supplemental Online Content

Rovati L, Zec S, Dziuba D, et al. Implementation of a multimodal knowledge-exchange platform to provide trauma critical care education during the ongoing conflict in Ukraine. *JAMA Netw Open.* 2023;6(2):e230050. doi:10.1001/jamanetworkopen.2023.0050

eFigure. Learning Needs Assessment Survey Results

eTable. Postsession Survey Additional Questions

This supplemental material has been provided by the authors to give readers additional information about their work.

© 2023 Rovati L et al. *JAMA Network Open.*

eFigure. Learning Needs Assessment Survey Results

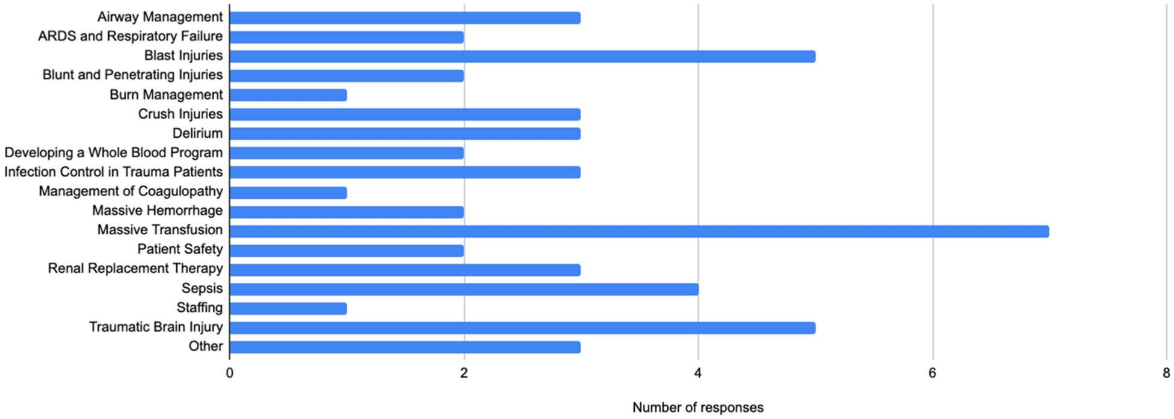

Topics of interest for tele-education sessions selected by participants. The data are based on 52 survey responses.

© 2023 Rovati L et al. *JAMA Network Open.*

eTable. Postsession Survey Additional Questions

Survey item	Responses No. (%)
**1. Objective 1: Define the importance of managing acutely ill and injured patients by the standardized, systematic and structured approach.**	**(n = 24)**
Met	18 (75%)
Partially Met	4 (16.7%)
Not Met	2 (8.3%)
**2. Objective 2: Incorporate the CERTAIN approach (admission checklist) to manage admission/resuscitation of acutely ill and injured patients.**	**(n = 24)**
Met	10 (41.7%)
Partially Met	12 (50%)
Not Met	2 (8.3%)
**3. Objective 3: Incorporate the CERTAIN approach (rounding checklist) to perform daily rounding for acutely ill and injured patients.**	**(n = 24)**
Met	11 (45.8%)
Partially Met	11 (45.8%)
Not Met	2 (8.4%)
**4. Objective 4: Demonstrate the prompter's role to provide real-time feedback to the team during the admission/resuscitation and rounding using the CERTAIN checklist.**	**(n = 24)**
Met	11 (45.8%)
Partially Met	8 (33.3%)
Not Met	5 (20.9%)
**5. Were evidence-based references incorporated when appropriate?**	**(n = 63)**
Yes	61 (96.8%)
No	2 (3.2%)

These questions were asked only after some of the webinars based on the presentation topic.

© 2023 Rovati L et al. *JAMA Network Open.*
