# Supplementary material for: Implementation of a Multimodal Knowledge-Exchange Platform to Provide Trauma Critical Care Education During the Ongoing Conflict in Ukraine
Source: JAMA Netw Open. 2023 Feb 10;6(2):e230050. doi: 10.1001/jamanetworkopen.2023.0050 (PMC9918882; doi:10.1001/jamanetworkopen.2023.0050)
Supplement: Supplement 2. — Data Sharing Statement [file jamanetwopen-e230050-s002.pdf]

Data Sharing Statement

Rovati. Implementation of a Multimodal Knowledge-Exchange Platform to Provide Trauma Critical Care Education During the Ongoing Conflict in Ukraine. *JAMA Netw Open*. Published February 10, 2023. doi:10.1001/jamanetworkopen.2023.0050

Data
Data available: Yes
Data types: Deidentified participant data
How to access data: The content on the website (https://www.icertain.org/ukraine) is freely available in Ukrainian, Russian and English languages. Analytics from secure ViberTM platform, SquarespaceTM and YouTubeTM will be provided upon request to researchers for the purpose of systematic reviews or related research projects.
When available: With publication

Supporting Documents
Document types: None

Additional Information
Who can access the data: Researchers whose proposed use of the data has been approved
Types of analyses: Systematic reviews or related research projects
Mechanisms of data availability: With investigator support
